# Effect of Type-2 Diabetes Mellitus on the Expression and Function of Smooth Muscle ATP-Sensitive Potassium Channels in Human Internal Mammary Artery Grafts

**DOI:** 10.3390/ph17070857

**Published:** 2024-07-01

**Authors:** Jovana Rajkovic, Miodrag Peric, Jelena Stanisic, Milos Gostimirovic, Radmila Novakovic, Vladimir Djokic, Snezana Tepavcevic, Jelena Rakocevic, Milica Labudovic-Borovic, Ljiljana Gojkovic-Bukarica

**Affiliations:** 1 Institute of Pharmacology, Clinical Pharmacology and Toxicology, Faculty of Medicine, University of Belgrade, 11000 Belgrade, Serbia; milos.gostimirovic@med.bg.ac.rs (M.G.); bukarica@rcub.bg.ac.rs (L.G.-B.); 2 Dedinje Cardiovascular Institute, 11000 Belgrade, Serbia; mperic2009@hotmail.com; 3 Laboratory for Molecular Biology and Endocrinology, Vinca Institute of Nuclear Sciences, University of Belgrade, 11000 Belgrade, Serbia; sjelena@vin.bg.ac.rs (J.S.); sradivojsa@vin.bg.ac.rs (S.T.); 4 Center for Genome Sequencing and Bioinformatics, Institute of Molecular Genetics and Genetic Engineering, University of Belgrade, 11000 Belgrade, Serbia; radmila.novakovic@imgge.bg.ac.rs; 5 Division of Nephrology and Hypertension, Mayo Clinic, Rochester, MN 55905, USA; djokic.vladimir@mayo.edu; 6 Institute of Histology and Embryology, Faculty of Medicine, University of Belgrade, 11000 Belgrade, Serbia; koskabg@gmail.com (J.R.); sborovic2001@yahoo.com (M.L.-B.)

**Keywords:** ATP-sensitive potassium channels, Kir6.1 subunit, human internal mammary artery, type-2 diabetes mellitus, pinacidil, vascular tone, spasm, coronary artery bypass surgery, bypass grafts

## Abstract

Here we have shown for the first time altered expression of the vascular smooth muscle (VSM) K_ATP_ channel subunits in segments of the human internal mammary artery (HIMA) in patients with type-2 diabetes mellitus (T2DM). Functional properties of vascular K_ATP_ channels in the presence of T2DM, and the interaction between its subunits and endogenous ligands known to relax this vessel, were tested using the potassium (K) channels opener, pinacidil. HIMA is the most commonly used vascular graft in cardiac surgery. Previously it was shown that pinacidil relaxes HIMA segments through interaction with K_ATP_ (SUR2B/Kir6.1) vascular channels, but it is unknown whether pinacidil sensitivity is changed in the presence of T2DM, considering diabetes-induced vascular complications commonly seen in patients undergoing coronary artery bypass graft surgery (CABG). K_ATP_ subunits were detected in HIMA segments using Western blot and immunohistochemistry analyses. An organ bath system was used to interrogate endothelium-independent vasorelaxation caused by pinacidil. In pharmacological experiments, pinacidil was able to relax HIMA from patients with T2DM, with sensitivity comparable to our previous results. All three K_ATP_ subunits (SUR2B, Kir6.1 and Kir6.2) were observed in HIMA from patients with and without T2DM. There were no differences in the expression of the SUR2B subunit. The expression of the Kir6.1 subunit was lower in HIMA from T2DM patients. In the same group, the expression of the Kir6.2 subunit was higher. Therefore, K_ATP_ channels might not be the only method of pinacidil-induced dilatation of T2DM HIMA. T2DM may decrease the level of Kir6.1, a dominant subunit in VSM of HIMA, altering the interaction between pinacidil and those channels.

## 1. Introduction

Cardiovascular diseases are the leading cause of mortality and morbidity in the world and represent a wide spectrum of diseases, including atherosclerosis and ischemic heart disease (IHD) [1]. In order to prevent chronic, debilitating and potentially irreversible complications of IHD, many patients often require cardiosurgical treatment as supplement to pharmacological therapy. Coronary artery bypass grafting (CABG) is a ground-breaking method developed in the middle of the last century which dramatically changed the treatment/outcome of patients with IHD. Today, it is one of the most common operative procedures in the world [2]. The technique involves the use of a healthy vascular conduit that would create a new path for blood to reach and supply cardiomyocites, avoiding damaged, narrowed or blocked blood vessel(s). Peri- and postoperative complications such as bleeding, infections, arrhythmias, stroke, kidney failure and cognitive dysfunction are more frequently seen in patients with co-morbidities, such as diabetes mellitus (DM) [3]. Those patients are also at higher risk for spasm of bypass grafts during and after CABG, and higher mortality [4,5]. 

For many decades now, the human internal mammary artery (HIMA) has been considered the ‘’gold standard’’ vascular conduit for CABG, and is still the best graft in clinical practice. The major advantage of HIMA over other blood vessels used for coronary revascularization, especially vein grafts, is its long-term patency. More than 90% of HIMA grafts are patent 10 years after the CABG, whereas less than 4% develop atherosclerosis, and only 1% have hemodynamic atherosclerotic stenosis [2]. Today, the left HIMA is preferred, and is called an “endocrine organ” [6]. However, its use is associated with some disadvantages as well, including its short length and the development of pre- (during the phase of graft preparation), peri- and postoperative (after the graft implantation) vasospasm [7]. Several factors may influence the development of graft spasm [8], for example physical (mechanical manipulation or temperature changes) or pharmacological (nerve stimulation or circulating vasoconstrictors such as norepinephrine, thromboxane A2, endothelin-1, serotonin (5-hydroxytryptamine, 5-HT) and angiotensin II, of which concentrations seem to be elevated during this procedure) [9]. Although the intact endothelial layer may act protectively and reduce the risk for short and long term graft spasms, its preservation in clinical practice is hard to achieve since it depends on several factors, such as surgical manipulation, a solution for blood vessel preservation, and the patient’s comorbidities, especially type-2 DM (T2DM) and metabolic syndrome [10]. In those conditions, over time, the endothelial layer loses its functionality and becomes less responsive to circulating vasoactive substances. This leads to impaired vascular tone and may be the ultimate reason for the progression of vascular spasms over time and eventual graft failure [11]. The endothelial dysfunction associated with prolonged hyperglycemia is of great clinical significance, considering the increased prevalence of T2DM among patients that require CABG [12]. Except T2DM, additional risk factors for endothelial dysfunction/graft failure include hypertension, hyperlipidemia, smoking and obesity, which all can be seen in patients undergoing CABG [13]. Therefore, it is important to investigate the vasoactive properties of the grafts and molecular mechanisms of their vasoreactivity in the population of patients with T2DM undergoing CABG, in order to predict or even prevent the aforementioned complications, which are notably higher in the presence of endothelial dysfunction. One of the most investigated mechanisms of vasoreactivity that does not include endothelium per se is associated with the activity of ion channels, notably potassium (K) channels [14,15].

K-channels are localized in different segments of blood vessels such as endothelium, vascular smooth muscle (VSM) and surrounding adipose tissue [16,17], and contain several subfamilies. In a healthy individual, those act synergistically in maintaining proper vascular tone and vasoreactivity. In vitro studies, however, have provided evidence that diabetic vascular dysfunction is associated with reduced smooth muscle K-channel activity [18,19], primarily ATP-sensitive K (K_ATP_) channels in VSM [20,21,22,23]. K_ATP_ channels are important regulators of vascular tone, and the changes in their expression or activity can be accompanied by an inadequate membrane repolarization, which leads to vasospasm [23]. Differences in functionality and the expression of K_ATP_ on the VSM of HIMA may explain the different vasodilatory response of HIMA from patients with and without T2DM. K_ATP_ channels in VSM are comprised of four Kir6.1 subunits and four SUR2B subunits, with only functional octameric complexes expressed on the plasmalemmal membrane of VSM. Similarly, Kir6.2 subunits in the heteromultimeric channel complex with SUR2B and Kir6.1 have been observed in human coronary artery endothelial cells [24]. The Kir6.x subunit has a pore-forming role, while SUR2B has a regulatory role and is the molecular target for sulfonylurea antidiabetics and potassium-channel openers (PCOs). 

PCOs are a diverse group of molecules which share a common tendency to activate K_ATP_ channels, and may be used as a well-defined pharmacological tool to investigate pharmacodynamics of this type of channel [25]. One of the most recognized PCOs is pinacidil whose mechanism(s) of action on the different blood vessels is well defined [26,27]. In their work, Gojkovic-Bukarica et al. had previously described the interaction between pinacidil and K_ATP_ channels on the segments of HIMA in patients with IHD but without T2DM [28]. The study brought evidence that pinacidil interacts with K_ATP_ channels via additional mechanism(s) of action which are not K channel- or endothelium-dependent. This imposed a need for further investigation and confirmation of this link in a model of HIMA segments with expected alterations in K_ATP_ channel expression and function, as can be seen in patients with T2DM [29]. Another study has shown that transgenic mice models of vascular spasm, with disrupted K_ATP_ channel subunits in VSM cells, spontaneously developed vascular spasm. The research conducted on Kir6.1-null mice showed a high rate of sudden death associated with Prinzmetal angina [30]. In the same research, pinacidil did not induce a K^+^ current in VSM cells obtained from Kir6.1-null mice and vasodilation was not produced. Also, in research on SUR2-null mice, a coronary artery vascular spasm developed, and despite restoration of the VSM K_ATP_ channels persistent spasm was confirmed [31]. All this points to the need to better study the expression, activity, and role of these channels in the vasoactivity of grafts from T2DM patients. 

Another important aspect of this issue is assessing the preoperative risk reduction of severe graft spasms by finding the best pharmacological antivasospastic agent for the solution in which grafts are preserved. So far, different methods were attempted such as prompt selective graft arteriogram or intraluminal injection of vasodilators (calcium antagonists, nitroglycerin) to relieve the vasospasm. Probably the best illustration for the necessity of studies like this can be found in clinical settings. In an immediate postoperative course after CABG, a patient with poorly controlled T2DM developed ventricular fibrillation due to hypokalemia, induced after required insulin infusions. This severe arrhythmia reduced the patient’s cardiac output, so he needed parenteral inotropic support (dopamine, dobutamine, adrenaline). Since HIMA has high expression of α-adrenergic receptors with little or no β-adrenoceptor function, the inotropic drugs caused severe vasospasm in the patient. Therefore, the inotropic agents (that saved the patient’s life) are potentially spasmogens in arterial grafts, which makes it difficult to reduce the risk for a complicated postoperative course [32]. At the same time, this highlights the need for underlining molecular mechanisms by which vasospasm could occur in complex, yet possible, clinical scenarios.

Therefore, the aims of the present study were:

1. to investigate the role of K_ATP_ channels in the pinacidil-induced vasodilation of HIMA from patients with T2DM
2. to evaluate whether rings of HIMA from patients with and without T2DM show differences in the expression of K_ATP_ channel subunits.

## 2. Results

### 2.1. Characteristics of Patients

Our study included 38 patients, with 21 diagnosed with T2DM. Nine patients were using insulin, while 12 patients were using only oral antidiabetic therapy. All patients on oral therapy used metformin. None of the patients on oral therapy used derivatives of sulphonylureas (including glibenclamide). Demographic and clinical characteristics of the included patients are shown in Table 1. Prescribed pharmacotherapy of these patients is represented in Table 2. 

### 2.2. Effects of Pinacidil and Glibenclamide on the Human Internal Mammary Artery Precontracted by Serotonin

**Figure 1:**
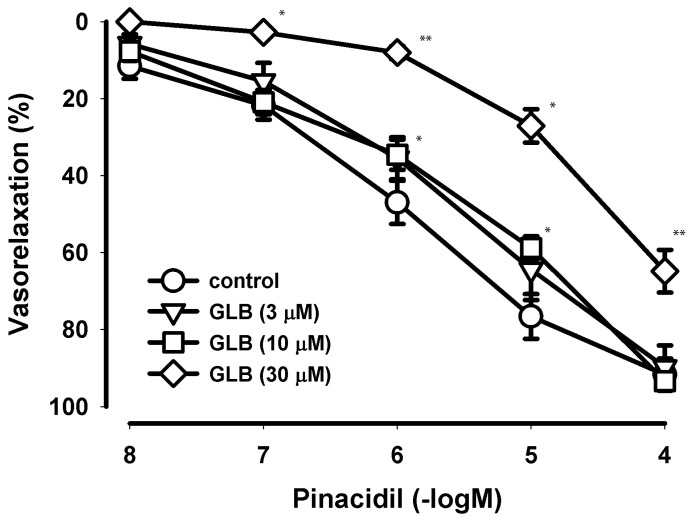
Concentration−dependent curves of pinacidil effects on the human internal mammary artery in the presence of blocker glibenclamide. The relaxant effect of pinacidil on the contraction provoked by the 5-hydroxytriptamine on the segments of the human internal mammary artery (HIMA) in type-2 diabetes mellitus (T2DM) patients in the presence of three different concentrations (3, 10 and 30 µM) of the selective blocker of K_ATP_ channels, glibenclamide (GLB). The points (circles, triangles, squares and diamonds) are the means and the vertical lines show the SEM (n = 10–14 for the controls and n = 5–7 in the presence of GLB; n—number of segments); * *p* < 0.05; ** *p* < 0.01.

Pharmacological experiments performed on HIMA segments from T2DM patients indicated that pinacidil produced vasodilation of HIMA rings (Table 3, Figure 1).

Glibenclamide (GLB), a highly selective blocker of K_ATP_ channels used in concentrations up to 10 µM did not affect the pinacidil-induced relaxation. However, in the high concentration (30 µM) which is not highly selective for K_ATP_ channels, GLB antagonized the effect of pinacidil.

GLB had no apparent effect on resting tension. The solvent of GLB (96% *v*/*v* ethanol) in all the used concentrations necessary for the prepared GLB (3–30 µM) solution had no apparent effect on resting tension and on the developed serotonin-induced tonic contractions (n = 14 all).

**Figure 2:**
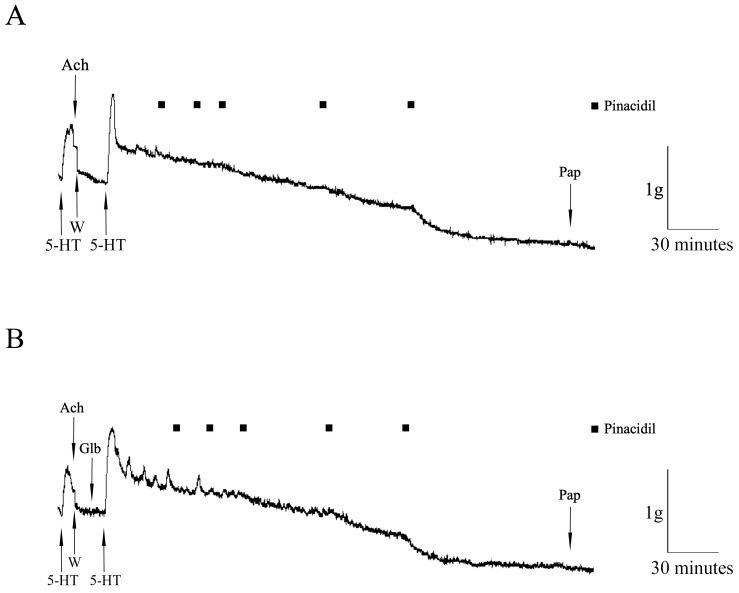
The original recordings of the pinacidil effects on the human internal mammary artery (HIMA) in T2DM patients. The effect of pinacidil (closed square, 0.01–100 µM) on the HIMA segments precontracted with 5-hydroxytryptamine (5-HT, 100 µM) in the absence (**A**) and in the presence (**B**) of glibenclamide (Glb, 3 mM). The presence of the endothelium was tested by adding acetylcholine (Ach, 100 µM); papaverine (Pap, 100 µM) was added as a general vasodilator; W—wash.

The original recordings of the pinacidil effects on the HIMA in T2DM patients precontracted with serotonin (in the absence and in the presence of GLB) are presented in Figure 2.

### 2.3. Molecular Analysis of K_ATP_ Channels: Immunohistochemistry

**Figure 3:**
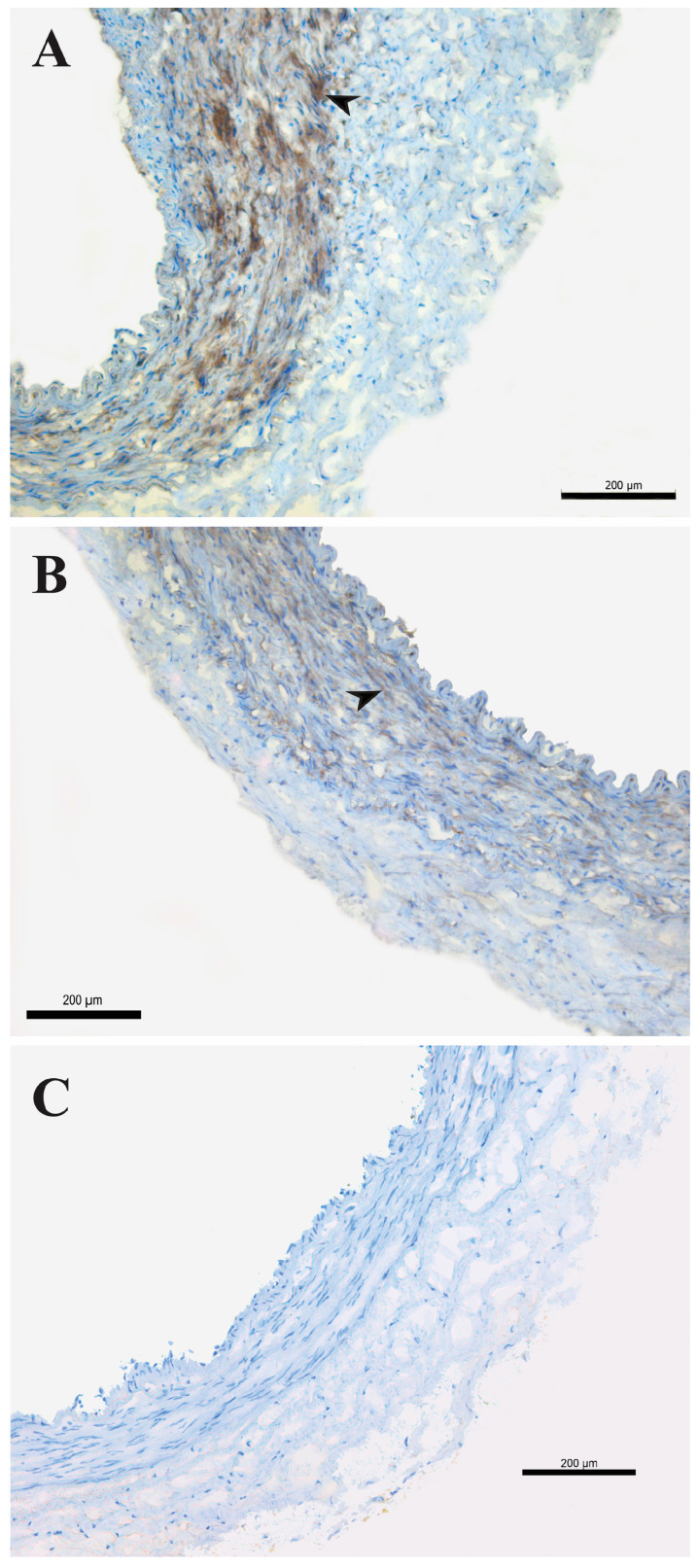
Detection of Kir6.1 antigens on the human internal mammary artery. Expression of Kir6.1 antigens on the human internal mammary artery (HIMA) from patients without diabetes mellitus (NDM, (**A**)) and with type-2 diabetes mellitus (T2DM, (**B**)) assessed by immunohistochemistry staining. Kir6.1 immunopositivity (brown staining, arrow) on the smooth muscle cells (**A**,**B**); negative control (**C**). Magnification 100×. Figure is representative of preparations from three patients.

VSM cells of HIMA showed Kir6.1 immunopositivity in both groups, predominantly with diffuse intracellular localization. HIMA obtained from non-diabetic patients showed moderate staining intensity (++ Figure 3A), compared to a weak (+) immunopositive signal in HIMA from T2DM patients (Figure 3B). HIMA samples from both NDM and T2DM showed no immunopositivity when applying antibodies for the Kir6.2 and SUR2B subunits. Previously, we reported with the same Kir6.2 antibody a lack of staining on a different tissue (human saphenous vein) [21].

### 2.4. Molecular Analysis of K_ATP_ Channels: Western Blot

**Figure 4:**
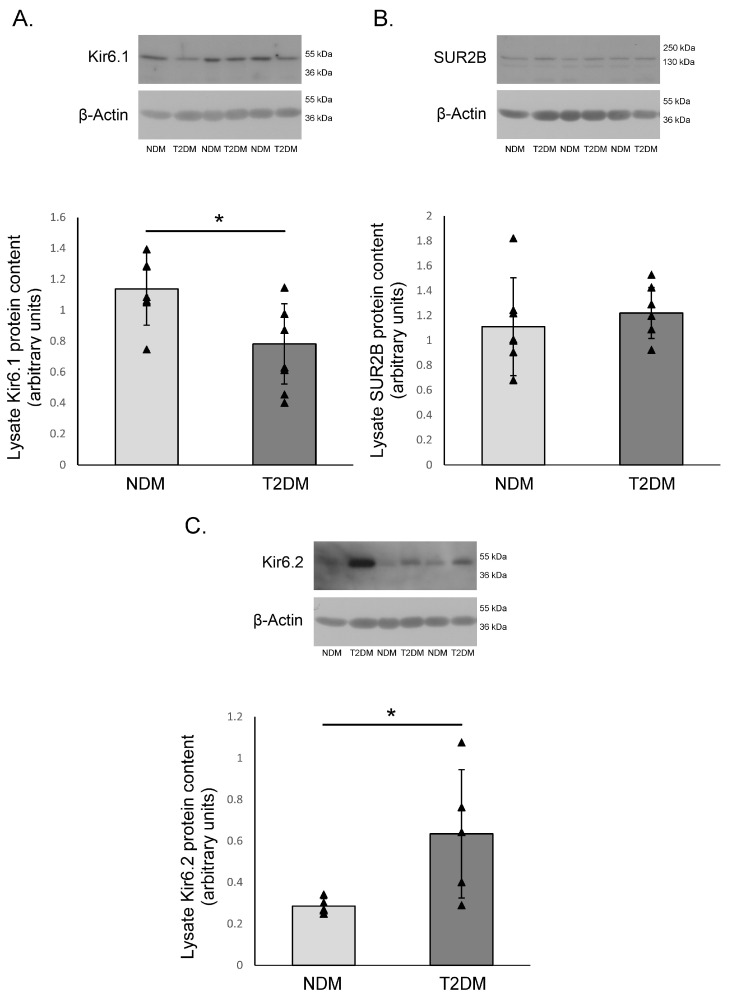
The protein expression of K_ATP_ channel subunits on the human internal mammary artery. The protein expression of the Kir6.1 (**A**), SUR2B (**B**) and Kir6.2 (**C**) subunits of the K_ATP_ channels on the human internal mammary artery (HIMA) from patients without diabetes mellitus (NDM) and with type-2 diabetes mellitus (T2DM). This figure presents mean values ± SD in 6 patients per group, all the bands show the samples from different patients. The values were normalized to β-Actin protein expression. * *p* < 0.05.

Using Western blot, all three subunits were detected. While there were no differences between SUR2B subunits among HIMA samples of patients with and without T2DM, Kir6.x subunits showed some variability. Kir6.1 subunit expression was significantly increased in HIMA segments obtained from the non-diabetic group compared to diabetic patients. Conversely, Kir6.2 was more highly expressed in diabetic patients (Figure 4.).

## 3. Discussion

This manuscript has shown for the first time the differences in the expression of Kir6.1, Kir6.2 and SUR2B subunits of K_ATP_ channels in HIMA from patients with T2DM. Also, we have shown that the blockade of K_ATP_ channels by a highly selective concentration of GLB (3 and 10 µM) on HIMA from patients with T2DM did not inhibit the endothelium-independent vasorelaxation caused by pinacidil, a known K_ATP_ channel opener. However, at a non-highly selective GLB concentration (30 µM), partial inhibition of the pinacidil effect was observed.

HIMA segments obtained after CABG were divided into two groups based on the presence of T2DM. The groups shared similar demographic and clinical characteristics. Interestingly, no differences in serum glucose levels, measured on the day of surgery, were observed. This may be due to the prediabetic phase of some NDM patients and their lack of regular glycaemia control. Patients with T2DM may also have a low compliance in the use of antidiabetic medication. However, prescribed pharmacotherapy was in alignment with the international guidelines for the treatment of the patients with IHD and T2DM [33]. Patients were carefully selected in order to avoid any potential influence of their pharmacotherapy to the results of this study. Although there are known gender differences in terms of the vascular reactivity of HIMA to different PCOs [34], outcomes after CABG [35] or even in the expression of cardiac K_ATP_ channels [36], there are no studies showing different expression of vascular K_ATP_ channel subunits between men and women. For that reason, the samples of HIMA from both sexes were included.

Pinacidil relaxes different human blood vessels, which we have previously described. The sensitivity (pD2) of the radial artery (6.09) was the highest, followed by the human saphenous vein (5.82), HIMA (5.77) and the human umbilical vein (4.57) [21,22,28,37]. Our results with samples without functional endothelium and/or patients with T2DM suggested that diabetes reduced the sensitivity of the human umbilical vein (4.29) to pinacidil, in contrary to the second most used bypass graft, the human saphenous vein (5.85). For this reason, we wanted to investigate the effects of diabetes on the most commonly used graft, HIMA. Interestingly, similar to the human saphenous vein (HSV), our results showed comparable sensitivity of HIMA from patients with and without T2DM (5.81 vs. 5.77). For this purpose, we used the middle pD_2_ among controls (comparing to 6.08 and 5.75, see results) because that concentration gave the maximal response (E_max_, 98%) compared to controls we used for lower doses of GLB (3 and 10 µM). Considering that diabetes did not change the sensitivity of HIMA to pinacidil and our previous reports of pinacidil’s action on NDM HIMA [28] and the radial artery [37], it made us think that pinacidil has additional mechanism(s) of relaxation in T2DM as well. This probably involves activation of different VSMC ion (predominantly subtypes of K) channels or acting via K-independent pathways (which will be the topic for our future research). Considering the well described influence of prolonged hyperglycemia/T2DM on the expression of K_ATP_ channels, we investigated how T2DM may alter relaxation properties of HIMA on the proposed PCO. Mechanisms of impaired K_ATP_ channel structure/function in VSMC during T2DM include overproduction of reactive oxygen species (ROS) and protein kinase C [38], and altered gene expression [39], which can all critically impact vascular tone/cause abnormal relaxation to both endogenous/exogenous vasodilators. In the grafts of our patients, this may increase postoperative sequelae (premature vasospasm) and contribute to worse outcomes after CABG. Also, considering the role/impaired expressions of K_ATP_ channels in the pathological conditions associated with hypoxia/ischemia/acidosis (which all can be seen in diabetes) and the previously described GLB-dependent effect of pinacidil to NDM HIMA, our main focus was pinacidil influence on the K_ATP_ channels in T2DM. Initial genetic studies of the relationship between pinacidil and K_ATP_ channels came in the early 2000s, from studies of two animal models: Kir6.1−/− and SUR2−/− mice. In both knockout models, there was no K_ATP_ channel activity in aortic VSMC and the vasodilating effect of pinacidil was abolished [30,40]. There is also evidence that pinacidil’s effects on K_ATP_ channels may be influenced by the intracellular concentrations of adenine nucleotides. Kir6.2/SUR2B can be activated by Mg-nucleotides (MgATP/MgADP) through binding with intracellular domains (NBFs) of the SUR subunit [41], thus, the interaction between [MgATP/MgADP]i and pinacidil may likely happen. In a study on *Xenopus leavis* oocytes, the presence of MgATP enhanced pinacidil’s affinity for the SUR2B subunit [42], which suggested that an altered nucleotide metabolism would have a permissive impact on the pinacidil sensitivity to the channel. Our future results on this model will further explain the cross-linking between PCOs and intracellular metabolic status.

In our previous research, 10 µM GLB partly inhibited relaxation of endothelium-denuded HIMA produced by a pinacidil analogue, P1075 [43]. Here, in the same (and lower, 3 µM) concentration, GLB failed to do so. However, in a higher concentration (30 µM), GLB succeeded in partially inhibiting pinacidil effects and the maximal response. There are two potential explanations for this concentration dependent effect. First, it was shown that GLB in high concentrations (>10 µM) has intracellular effects, such as blocking the mitochondrial K_ATP_ channels (mito K_ATP_) and modulating/decreasing intracellular Ca^2+^ turnover [44]. Mito K_ATP_ subunits (pore-forming mitoKir (CCDC51) and the regulatory subunit mitoSUR (ABCB8)) are known to be activated by some PCOs as well [45]. Second, at the high concentrations (>10 µM), GLB may act via K_ATP_-independent effects, and activate other types of membrane K-channels: (voltage-gated (Kv) and Ca^2+^-activated (K_Ca_) channels, 10–100 µM), Na/Ca exchanger (NCX, 10 µM), Na/K pump (200 µM) and/or L-type Ca^2+^ channels (200 µM) [37,46]. Additionally, T2DM is often accompanied with metabolic acidosis, which changes the vascular reactivity of blood vessels [47]. Relaxation of HIMA happens to be enhanced in an acidic environment (pH 6.8) [48], which might influence our results too. However, we did not collect blood-gas analyses (ABG) from patients, and cannot confirm whether they were in acidosis. Second, a pH of 6.8 represents severe acidosis, which is “hallmark” of diabetic ketoacidosis, more commonly seen in type-1 DM (T1DM).

The correlation between the impaired functional response to pinacidil and the reduced expression of K_ATP_ channels in the presence of (gestational) diabetes mellitus (gDM) was shown in a human umbilical vein (HUV) model [22]. Interestingly, the same finding on IHC/WB was shown in the separate experimental group, a population of women with both gDM and hypertension (HTA), but pinacidil-induced vasodilation remained intact. This was explained by the compensatory increase in the functional BK_Ca_ channels. Patients in our study (especially in the T2DM group) however, have overlapping T2DM and HTA, and there are no separate groups of patients with exclusively one disease. The inability to exclude the influence of HTA on our finding represents another limitation of our study. However, in clinical practice, the number of patients with solely one disease (and the indication for CABG) is very limited, considering the multifactorial etiology of CVD and the increasing prevalence of cardiometabolic diseases everywhere in the world, including in the Republic of Serbia [49]. 

A major finding of our study is the expression patterns of different vascular K_ATP_ channel subunits. So far, Kir6.1 expression has been found in different tissues, predominantly throughout the vasculature [50]. The study on transgenic mice showed that VSMC K_ATP_ currents were absent in Kir6.1−/− mice, resulting in a hypertensive phenotype [51]. Wang et al. found decreased cardiac function and Kir6.1 expression in mice with diabetic cardiomiopathy [52]. This was associated with the aggravation of cardiac dysfunction. On the contrary, Kir6.1 overexpression improved cardiac function, both in vivo and in vitro. As for the Kir6.2 subunit, gain-of-function (GOF) mutations (KCNJ11/ABCC8) in the Kir6.2 subunits of pancreatic K_ATP_ channels have been identified as the most common cause of human neonatal diabetes mellitus, nDM [53]. This form of diabetes is rarely present in newborns but very often symptoms start in late infancy (six months or older) [54]. Based on the age of our patients, the number of them receiving insulin (42.9%), as well as the lack of exact genetic studies, we cannot exclude two possible explanations for our results. Our patients could share similar mutations of the vascular K_ATP_ channels, causing vascular tone alteration. Alternatively, prolonged hyperglycemia could be the cause for the altered Kir6.2 subunit expression and variable response to pinacidil, and the concentration-dependent effects of GLB. 

In endothelium-denuded segments of aorta from Wistar-Kyoto (WKY) spontaneously hypertensive rats (SHR), the expression of VSMC Kir6.1 and SUR2B subunits were confirmed, while Kir6.2 was not detected [55]. Park et al. reported decreased expression of Kir6.1 and SUR2B subunits in the hypertrophied aortic smooth muscle cells of rabbits [56]. Also, decreased expression of Kir6.1, Kir6.2 and SUR2B was observed in the human umbilical arterial smooth muscle cells obtained from women with gDM [57]. We have shown by IHC that Kir6.1 subunit proteins are present on the VSMC of both NDM and T2DM HIMA. However, we failed to detect the Kir6.2 subunit in both groups of HIMA by using the Kir6.2 antibody mentioned in the Section 4. In our previous research on HSV we reported the same limitation using antibodies from the same manufacturer [21]. However, in earlier research done in our laboratory both pore-forming subunits (Kir6.1 and Kir6.2) were detected on HIMA by IHC [43]. 

Recently, there are studies on advancements in the treatment of vascular complications in T2DM, for example by using pulsed magnetic field (PMF) [58]. Interestingly, this novel approach had an impact on the K_ATP_ channel expression in a model of aortic rings from diabetic rats, where PMF inhibited both Kir6.1 and Kir6.2 mRNA expression. Another in vitro study suggested the inhibition of Kir6.2 expression by a novel anti-diabetic drug, mitiglinide [59], and Kir6.2/SUR2B expression by a novel substance, PNU-37883A [60]. However, in our case, none of the patients used therapy (oral sulphonylureas) that could interfere with the results obtained from WB, thus any bias regarding pharmacotherapy influence is unlikely.

The expression of K_ATP_ channel subunits is differentially changed by diabetes (Kir6.1 is reduced, SUR2B is not changed and Kir6.2 is increased), but the relaxation of HIMA to pinacidil was not changed. It is possible that pinacidil has additional mechanism(s) of action, independent of K_ATP_ channels. But there are other endogenous ligands that open vascular K_ATP_ channels as a compensatory mechanism to patophysiological conditions [61]. K_ATP_ channels are “metabolic sensors”, and their expression and function are of vital interest to the adaptation of blood vessels to pathophysiological conditions. So further research is necessary to investigate how altered expression of K_ATP_ subunits in T2DM influence the relaxation of HIMA to endogenous ligands mentioned before. 

In the end, we want to highlight the importance of similar research in the future, mostly due to its enormous clinical significance. Identification of an ideal vasodilator (solution) that can prevent the onset/reverse of spasms will surely reduce peri/postoperative sequelae and mortality long-term after CABG. 

## 4. Materials and Methods

Specimens of HIMA were collected after CABG interventions. The CABG procedures were performed at the Institute for Cardiovascular Diseases ‘’Dedinje’’ (Belgrade, Serbia). Small vials filled with Krebs-Ringer bicarbonate (KRb) solution were used for the transportation of the samples to the Laboratory for Cardiovascular Pharmacology, Faculty of Medicine, where they were stored at a temperature of 4 °C. Immediately after surgical removal, samples of HIMA were used for the pharmacological experiments. In the case of non-pharmacological experiments—Western blot and immunohistochemistry, right after the surgical procedure samples were promptly snap-frozen using liquid-nitrogen and kept at −80 °C. All samples of HIMA represented unused parts of the blood vessel after the CABG surgery and intraoperative manipulations. Variables of great significance for the study were collected: gender, age, body mass index (BMI), smoking habits, presence of hyperlipoproteinemia and hypertension, presence and type of diabetes mellitus and pre-operative chronic therapy. Patients receiving GLB or other sulphonylureas were excluded from the study.

### 4.1. Pharmacological Experiments

Samples of HIMA were cut in small segments approximately 3 mm long. The surrounding tissue was cut from the rest of the samples and the endothelial layer was mechanically removed by gentle scraping with a steel wire. The procedure constituted the following steps. Through the lumen of the proposed blood vessels, two miniature steel triangles were inserted and mounted into the system for isolated organs. The lower triangle was hooked to the tube at the bottom of the 10-mL volume organ bath, while the upper triangle was hooked to the transducer via the string of the triangle. Isometric contractions of the samples were measured using K30, Hugo Sachs, Freiburg, Germany system, while the IsoLAB software (v2.0, Elunit, Belgrade, Republic of Serbia) was used for data recording. During the 45 min incubation period, at a temperature of 37 °C and pH of 7.4, organ bath-containing solutions were washed every 15 min using KRb solution with the following content: NaCl 120 mM, KCl 5 mM, MgSO_4_ 1.2 mM, CaCl_2_ 2.5 mM, KH_2_PO_4_ 1.2 mM, NaHCO_3_ 25 mM and glucose 11 mM. The solution was then exposed to the gas mixture: 95% O_2_ and 5% CO_2_. Before the beginning of the experiment, every segment was progressively tightened to the optimal tension point at which the basal tone was reached (3 g in HIMA). The endothelium of the HIMA segments were removed during the preparation of HIMA. For every sample, the presence of endothelium was checked by applying acethylcholine (100 µM) immediately after the submaximal contraction induced by 5-hydroxytriptamine (5-HT, 100 μM) was reached [62]. The absence of the acethycholine-induced vasorelaxation of the HIMA suggested a lack of endothelium, thus enabling the determination of the pinacidil’s effect only in the vascular smooth muscle cells. All experiments were divided into different groups according to the concentrations of the applied blocker. Each group constituted 6–8 single experiments. Different segments of each HIMA were used for different series. For every experiment an adequate control group was provided, i.e., samples of each patient were divided into experimental and control groups, according to the aforementioned criteria. 5-HT (100 µM) was used as a vasoconstrictor. After achieving the stable tone, pinacidil was added in cumulative, increasing doses (0.01–100 µM). When the effect of the highest concentration of pinacidil (100 µM) was achieved, papaverine (100 µM), was added as a general blood vessel dilatator. To determine the involvement of K_ATP_ channels in the pinacidil-induced vasorelaxation, glibenclamide (GLB, 3, 10 and 30 µM), a specific blocker of these channels, was used. GLB had been applied 10 min before accomplishing 5-HT-induced contraction. For the interpretation of the effects of pinacidil, the percentage of the maximal potential relaxation caused by papaverine was calculated. The experiments were graphically presented in a multiple curve design.

### 4.2. Immunohistochemical Analysis

Sections (10 µm thick) (Cryostat Leica CM1850, Leica, Deer Park, IL, USA) of HIMA frozen samples were serially cut and fixed in a 1:1 mixture of the methanol and acetone (10 min at −20 °C), and washed in the TBS (Tris-Buffered Saline). After the fixation, all the steps were carried out at room temperature in a humidified chamber. In order to block non-specific staining, the slides were incubated in the 1.5% normal blocking serum in the PBS (Phosphate-Buffered Saline) for one hour. Overnight incubation was performed with the following primary antibodies: anti-Kir6.1 (dilution ratio 1:50), anti-Kir6.2 (dilution ratio 1:50) and anti-SUR2B (dilution ratio 1:50). The tissue sections incubated with the rabbit primary antibody were treated by applying the commercial UltraVision/DAB staining kit (Thermo Scientific LabVision TL-060-HD, Rockford, IL, USA). Briefly, in order to reduce nonspecific background staining due to endogenous peroxidase, slides were incubated with Ultravision Hydrogen Peroxide Block for 10 min. Afterwards, additional nonspecific background staining was blocked by the incubation of slides with Ultravision Protein Block for 5 min. After the incubation with the primary antibody, Primary Antibody Enhancer was applied and incubated for 10 min, with subsequent incubation with HRP Polymer for 15 min. Visualization of immunopositivity was achieved by using a mixture of DAB Quanto Chromogen and DAB Quanto Substrate. After each step, slides were washed in buffer (PBS). Sections incubated with the goat primary antibodies were treated by applying the commercial ImmunoCruz™ goat ABC Staining System (sc-2023, Santa Cruz Biotechnology, Inc., Dallas, TX, USA). Briefly, after the overnight incubation with the primary antibody, sections were incubated with the biotinylated secondary antibody for 1.5 h, followed by 30 min incubation with AB enzyme reagent and subsequent incubation of slides in 1–3 drops of peroxidase substrate until the desired stain intensity was achieved. After each step, slides were washed in buffer. Negative controls were obtained by omission of the primary antibody. All samples were counterstained by Mayer’s hematoxylin for 120 s. All histological slides were analyzed using the Leica DM4000 B LED microscope (Leica, Wetzlar, Germany) with the digital camera Leica DFC295 (Leica, Heerbrugg, Switzerland), using the Leica Application Suite (LAS, v4.4.0) software system [63].

Immunopositivity was estimated by using the semi-quantitative method, independently by two histologists unaware of the experimental groups. Intensity of immunopositivity was graded into four categories: negative (−), weak (+), moderate (++), and intensive (+++).

### 4.3. Western Blot

The fresh frozen HIMA segments (6 patients per group) were homogenized on ice with an Ultra-turrax homogenizer (3 × 30 s) in ten volumes (m:V) of modified RIPA buffer (50 mmol/L Tris/HCl, pH 7.4, 150 mmol/L NaCl, 1% Triton X-100, 0.2% Na-deoxycholate, 0.2% SDS, 1 mmol/L EDTA, protease and phosphatase inhibitors). After homogenization, tissue samples were centrifuged for 30 min, at 11,200 rpm and 4 °C. After supernatant had been made, it was used as a cell lysate. In order to quantify total protein in the tissue sample, protein concentrations were registered at 562 mm using a spectrophotometer and the bicinchoninic acid protein kit assay (SERVA Electrophoresis, Heidelberg, Germany). Bovine serum albumin (BSA) (25–1000 μg/mL) was used for the creation of a calibration curve. Protein samples were desaturated by adding a 2× Laemli buffer, proteins were boiled for 5 min at 95 °C and after that SDS-PAGE electrophoresis in a 10% polyacrylamide gel was made. Proteins (30 μg per line) separated by electrophoresis were switched to the polyvinylidene fluoride membranes (Merck Millipore Ltd., County Cork, Ireland) by wet transfer and then blocked with 5% bovine serum albumin for 90 min. All membranes were incubated overnight at 4 °C with specific primary antibodies anti-Kir6.1 (sc-20808, with dilution of 1:1000), antiKir6.2 (sc-11228, with dilution of 1:500) and anti-SUR2B (sc-5793, with dilution of 1:500). All primary antibodies were purchased from the Santa Cruz Biotechnology, Inc. company. After profound washing (5 × 5 min) with TBST buffer (10 mM Tris-HCl pH 7.5, 100 mM NaCl, 0.02% Tween 20), the secondary horseradish peroxidase (HPR)-conjugated antibodies in 1:10,000 dilution (Santa Cruz Biotechnology, Inc. company) were applied on the membranes and left for 90 min at room temperature. After membrane washing (5 × 5 min), an enhanced chemiluminescence method was used for protein visualization according to the manufacturer’s instructions (Sigma-Aldrich, Saint-Louis, MO, USA). After that, the stripping buffer (100 mmol/L), consisting of β-mercaptoethanol, 2% SDS and 62.5 mmol/L Tris (pH 6.7), was used for stripping the membranes at a temperature of 50 °C for a period of 0.5 h. Stripped membranes were washed 2 × 10 min with TBST, blocked with 5% BSA for 90 min, and blotted with β-Actin antibody (sc-1616, Santa Cruz Biotechnology, Inc. Company, with a dilution of 1:1000) as a control for protein input. The values were normalized to β-Actin protein expression. Images were scanned and quantified using specific software—ImageJ (v1.51, NIH, Bethesda, MD, USA) [64].

### 4.4. Drugs and Solutions

In pharmacological experiments the following drugs were used: pinacidil, 5-HT, acetylcholine, papaverine and GLB, all obtained from the Sigma—Aldrich Inc., St. Louis, MO, USA. Pinacidil was dissolved in distilled water (with the addition of few drops of 0.1NHCl), GLB in the 96% *v*/*v* ethanol, while other substances were dissolved in distilled water. All drugs were added directly to the bath in a volume of 100 µL, and the given concentrations represent definitive concentrations in the bath solution. Solvents of pinacidil and GLB did not change basal tone and the developed serotonin-induced tonic contraction (n = 4, both).

All primary antibodies (anti-Kir6.1, anti-Kir6.2 and anti-SUR2B) were purchased from Santa Cruz Biotechnology, Inc.

### 4.5. Statistical Analysis

For each experiment the EC_50_ (effective concentration producing 50%-maximum effects) was calculated by linear regression analysis and expressed as pD_2_ (−logEC_50_). The results were tested for normality and data were presented as the mean ± standard error of the mean (SEM); n refers to the number of experiments. In order to test the significance of differences for the pharmacological experiments and for the Western blots, the Student’s *t*-test was used; *p* < 0.05 was considered statistically significant. The graphic was designed in SigmaPlot (v14.0, Systat Softwar Inc., San Jose, CA, USA), while all calculations were done in the GraphPadPrism (v9.4.1, GraphPad Software Inc., San Diego, CA, USA).

## 5. Conclusions

Herein we have indicated that T2DM impairs dilatation of HIMA by decreasing the expression and function of K_ATP_ channels. More precisely, by changing the expression of different VSMC K_ATP_ channel subunits. However, the fact that pinacidil (which is PCO) were able to relax HIMA of T2DM patients, probably via additional K_ATP_ channel-independent mechanisms of action, provides an opportunity to investigate the effects of other substances with similar vasodilator effects on the impaired/damaged endothelium, as seen in diabetic patients undergoing CABG. More importantly, these results may have clinical significance, since the different expression and function of K_ATP_ channels in HIMA from patients with and without T2DM may play an important, major role in choosing/defining the adequate type of graft for patients with T2DM, especially in the presence of vascular complications like HTA or the occurrence of peri- and postoperative vasospasm, caused by impaired glucose regulation. Based on this, we could more precisely predict the functionality of the chosen/implemented graft and its patency many years after surgery. Also, post-operative antidiabetic pharmacotherapy for diabetes may be adjusted in patients with T2DM based on a decrease in the number of functional K_ATP_ channels, and consequently reduced capability for vasodilation. However, this study has certain limitations. First, the sample size should be increased in both groups and more detailed information about quantitative differences in K_ATP_ channel subunits should be obtained by PCR. Further, duration of the T2DM (and HTA) was not noted and we did not have the access of the radiological findings (e.g., Color Doppler of the blood vessels and other radiological assessments), thus we were not able to consider the impact of morphological and morphometric changes in the microvasculature (except those seen in the IHC). Since the patients were mostly receiving polytherapy, the impact of the treatment on measured parameters was not discussed. Hence, for the final conclusion further investigation should be done.

## Figures and Tables

**Table 1:** Patient demographic characteristics.

Patient Demographic Characteristics	NDM (n = 17)	T2DM (n = 21)	*p*
Male/Female	16/1	18/3	
Age (±SD, years)	63.9 ± 6.6	66.8 ± 7.6	*p* > 0.05
Smoking - current - former - never	6 7 4	2 11 8	*p* > 0.05
Hypertension	14	21	*p* > 0.05
Hyperlipoproteinemia	15	20	*p* > 0.05
BMI (±SD)	28 ± 4.1	28.4 ± 3.8	*p* > 0.05
Glycaemia (±SD, mmol/L)	6.7 ± 2.1	7.9 ± 2.3	*p* > 0.05

NDM—non diabetes mellitus patients; T2DM—type 2 diabetes mellitus patients; n—number of patients; SD—standard deviation; BMI—body mass index; glycaemia (mmol/L) measured on the day of surgery.

**Table 2:** Patient’s therapy prior to surgery.

Therapy Prior to Surgery	NDM (n = 17)	T2DM (n = 21)
ACE inhibitors (n, %)	7 (41.2%)	16 (76.2%)
β blockers (n, %)	9 (52.9%)	16 (76.2%)
CCBs (n, %)	2 (11.8%)	6 (28.6%)
Diuretics (n, %)	4 (23.6%)	6 (28.6%)
Statins (n, %)	8 (47.1%)	7 (33.3%)
Antiplatelet drugs (n, %)	12 (70.6%)	11 (52.4%)
Vasodilators (n, %)	5 (29.4%)	11 (52.4%)
Anticoagulants (n, %)	2 (11.8%)	5 (23.8%)
Antidiabetics - Oral (metformin) - Insulin	0 0	14 (66.7%) 9 (42.9%)

NDM—non diabetes mellitus patients; T2DM—type-2 diabetes mellitus patients; n—number of patients; CCB—calcium channels blockers.

**Table 3:** The effects of pinacidil on the human internal mammary artery in the presence of blocker glibenclamide.

	Type 2 Diabetes Mellitus (T2DM)	
	pD_2_ ± SEM	Emax (%) ± SEM
control	5.75 ± 0.50	90.47 ± 9.53
GLB (3 µM)	5.53 ± 0.30	89.76 ± 5.64
control	6.08 ± 0.37	89.18 ± 7.24
GLB (10 µM)	5.67 ± 0.10	93.45 ± 2.05
control	5.81 ± 0.10	98.03 ± 1.97
GLB (30 µM)	3.88 ± 0.36 *	64.84 ± 5.55 **

The effects of pinacidil on the contractions of the human internal mammary artery (HIMA) segments of type-2 diabetes mellitus (T2DM) patients provoked by 5-hydroxytriptamine (100 μM) in the absence (control) and in the presence of the K_ATP_ channels specific blocker, glibenclamide (GLB); results are presented as the mean ± SEM; n = 5–7 T2DM; n—number of segments per group; * *p* < 0.05; ** *p* < 0.01.

## Data Availability

The original contributions presented in the study are included in the article, further inquiries can be directed to the corresponding author.
